# Follicular dendritic cells display microvesicle-associated LMP1 in reactive germinal centers of EBV+ classic Hodgkin lymphoma

**DOI:** 10.1007/s00428-019-02605-w

**Published:** 2019-06-15

**Authors:** Stefania Uccini, Mazin F. Al-Jadiry, Giuseppina Pepe, Anna Pasquini, Adel R. Alsaadawi, Salma A. Al-Hadad, Arianna Di Napoli, Claudio Tripodo, Luigi Ruco

**Affiliations:** 1 Istituto Pasteur Italia, Rome, Italy; 2 Children’s Welfare Teaching Hospital, Baghdad College of Medicine, Baghdad, Iraq; 3 Department of Clinical and Molecular Medicine, Pathology Unit, Sant’Andrea Hospital, Sapienza University, Via di Grottarossa 1035, 00189 Rome, Italy; 4 Department of Pathology, Baghdad Medical City Complex, Baghdad, Iraq; 5 Tumor Immunology Unit, University of Palermo, Palermo, Italy; 6 Tumor and Microenvironment Histopathology Unit, the FIRC Institute of Molecular Oncology, Milan, Italy

**Keywords:** Latent membrane protein-1 (LMP1), Epstein-Barr virus (EBV), Classic Hodgkin lymphoma (cHL), Follicular dendritic cells (FDCs), Programmed death ligand 1 (PD-L1), Exosomes and microvesicles

## Abstract

Expression of the latent membrane protein-1 (LMP1) of Epstein-Barr virus (EBV) was investigated in 153 cases of EBV+ classic Hodgkin lymphoma (cHL); 120 cases were pediatric patients (< 14 years of age) from Iraq, and 33 cases were adult patients from Italy. We describe for the first time the presence of LMP1 protein in EBV-encoded RNA (EBER)-negative follicular dendritic cells (FDCs) of reactive germinal centers (GC) associated with EBV+ cHL. Presence of LMP1+ GCs was independent of geographic region and age of patients. Variable numbers of reactive GCs were present in 22.2% of cases (34 of 153), whereas LMP1 staining of FDCs was present in about a third of cases (10 of 34) with reactive GC. Most cases with LMP1+ GC were mixed-cellularity (MC) subtype, but some nodular sclerosis (NS) was also present. GC cells with LMP1+ FDCs were surrounded by numerous EBV-infected cells which were positive for EBER, LMP1, and CD30. Double immunolocalization analysis revealed that LMP1 was associated with CD63, an exosomal marker, and with CD21. The possibility is discussed that peri-follicular EBV-infected cells release LMP1 protein, perhaps through exosomes, and that the protein is then captured by FDCs and is presented to EBER-negative GC B cells.

## Introduction

EBV generally establishes different forms of latency, depending upon the phenotype and the transcription factor repertoire of the infected cell [1]. Under pathological conditions, the viral latent gene expression varies in different tumors [2]. The viral latent gene expression observed in nasopharyngeal carcinoma (NPC) and Hodgkin lymphoma (HL) is of intermediate type II latency and is characterized by expression of latent membrane protein-1 (LMP1) in tumor cells. In fact, using immunohistochemistry, it was demonstrated that LMP1 is present in Hodgkin/Reed-Sternberg (HRS) cells of EBV+ classic Hodgkin lymphoma (cHL) [3, 4]. EBV infection precedes HRS cell transformation. When present in the host cell, LMP1 acts as a mimic of CD40 and may contribute to the survival of HRS cell precursors by constitutively activating several of the pathways, including NF-kB, JAK/STAT, and phosphatidylinositol3-kinase/AKT, known to be aberrantly activated in HRS cells [2]. Localization of LMP1 to peri-nuclear regions of the cell is believed to be necessary to mediate these signaling functions [5].

LMP1 has also been demonstrated to localize to internal Golgi and multi-vesicular bodies (MVB). In lymphoblastoid cell lines, LMP1 was shown to be released into the extracellular spaces, possibly associated with exosomes [6–8]. Exosomes are a population of small (40–150 nm) endocytically derived extracellular vesicles [9]. They are produced from inward budding events on the limiting membrane of late endosomal organelles, forming intra-luminal vesicles in MVB. Similar to mechanisms of egress used by viral particles, MVBs can fuse with the plasma membrane to release exosomes into the extracellular space [9]. In further studies, it was shown that LMP1 is co-localized with the late endosomal protein Lamp3/CD63 [10] and that CD63 has a role in regulating LMP1 exosomal packaging and vesicle production [9].

Exocytosis of MVB and the release of exosomes were previously described in B and T lymphocytes and macrophages. It was shown that preparations of LMP1-containing exosomes have an immunosuppressive effect since they inhibit proliferation of peripheral blood mononuclear cells. Moreover, it was suggested that exosomes containing LMP1 could exert an anti-proliferative effect in cHL, thus allowing tumor cells to evade the immune system [10].

In the present report, we describe 10 cases of EBV+ cHL in which LMP1 protein was present in HRS cells and in CD21+/EBV-encoded RNA (EBER)-negative follicular dendritic cells (FDC) of reactive germinal centers (GC) as well. Furthermore, co-localization of LMP1, CD63, and CD21 could be demonstrated. Our findings are consistent with the possibility that LMP1 is released by EBV-infected cells located around follicles, possibly via exosomes, and is eventually captured by FDCs of GCs.

## Materials and methods

Formalin-fixed paraffin-embedded (FFPE) sections of 153 cases of EBV+ cHL were investigated for expression of LMP1 protein (Table 1). The majority (120 cases) were pediatric (< 14 years of age), first diagnosed at the Children’s Welfare Teaching Hospital of Baghdad College of Medicine, Iraq, and then sent for a second opinion to the Pathology Unit of Sant’Andrea Hospital of Rome, Italy [11]. Relevant clinical data were available for all the cases. None of the children had clinical or laboratory evidence of immunodeficiency. Results obtained with EBV+ cHL of Iraqi children were compared with those of 33 Caucasian adult patients diagnosed in Rome, Italy. The study was performed in accordance with the Helsinki Declaration and was approved by the local (Iraq) Ethical Committee. In Italy, Institutional Review Board was obtained (EC no. 168/SA/2003).

**Table 1:** EBV+ cHL with LMP1+ reactive GCs

Patients	n.	Mean age ± SD	M:F	Histology subtype	GC+ histology	GC+/LMP1+ histology
Children < 14 years of age*	120	7.6 ± 2.9	3.6:1	68 MC (57%) 52 NS (43%)	27/120 (23%)** 17 MC (63%) 10 NS (37%)	8/120 (6.7%)** 7 MC (88%) 1 NS (12%)
Adults > 14 years of age*	33	53.5 ± 16.8	2:1	22 MC (67%) 11 NS (33%)	7/33 (21%)** 5 MC (71%) 2 NS (29%)	2/33 (6%)** 1 MC (50%) 1 NS (50%)
Total number	153			90 MC (59%) 63 NS (41%)	34/153 (22%)**	10/153 (6.5%)**

*Pediatric cases were from Iraq; adult cases were Caucasian patients from Italy

**Number of positive cases/number of investigated cases

### Immunoperoxidase staining

Paraffin sections were immunostained for CD30 (UCS Diagnostics, Italy), CD3, CD20, (Novocastra, UK), CD79a, CD15, CD68, CD21, LMP1 (mouse clones CS1–4, IgG1 kappa), CD63, PD-L1 (22C3), and PD1 (DAKO, Denmark), PAX5 (Thermo Scientific, USA), using an automated immunostainer (OMNIS, Agilent, USA). The anti-LMP1 CS1–4 mAb is a widely used reagent to detect LMP1 protein in paraffin sections. CS1–4 was originally developed by Rowe et al. [12] from mice immunized with a beta-galactosidase fusion protein containing the carboxyl half of the B95.8 strain LMP sequence. We have controlled the specificity of CS1–4 with a pre-absorption experiment. CS1–4 mABs (.023 mg/mL) were pre-incubated with a 10–20-fold higher concentration of recombinant EBV-LMP1 protein, partial AA 185–366 (MyBioSource, California, USA) that contain the sequence used to generate CS1–4. Results indicated that pre-incubation with recombinant LMP1 protein was effective in abolishing the staining for LMP1 of FDCs and of EBV-infected HRS cells. Furthermore, the specificity of CS1–4 for EBV-LMP1 protein was supported by the presence of LMP 1+ cells only in cases of EBV+ cHL (EBER+) and of EBER+ nasopharyngeal carcinoma; cases of cHL negative for EBV (EBER-negative) were consistently negative also when immunostained with CS1–4.

In situ hybridization for EBER was performed on paraffin sections using Epstein-Barr Virus (EBER) PNA Probe/Fluorescein and FITC/HRP (DAKO, Denmark).

### Immunofluorescence staining

Double-marker immunofluorescence analysis was performed on 4-μm-thick FFPE tissue sections following deparaffinization and heat-based antigenic retrieval as previously reported [13]. The sections were incubated overnight at 4 °C with the following primary antibodies: monoclonal anti-human LMP1 (clone CS1/CS4, 1:100 pH 9, Novocastra), monoclonal anti-human CD30 (clone Ber-H2, 1:20 pH 9, DAKO), monoclonal anti-human CD63 (clone EPR5702, 1:1000 pH 9, Abcam), polyclonal anti-human CD21 (760–4438, Ready-to-use, pH 9, Ventana). Bound antibodies were revealed by the following fluorochrome-conjugated secondary antibodies: Alexa Fluor 568 goat anti-mouse IgG (H+L) (1:300 dilution) and Alexa Fluor 488 goat anti-rabbit IgG (H+L) (1:250 dilution) both from Invitrogen Molecular Probes. Nuclei were counterstained with DAPI (4′,6-diamidin-2-fenilindolo). For double-marker immunofluorescence stainings in which primary antibodies of the same made were adopted, the tyramide signal amplification system Opal Multiplex IHC kit (lot number 2395285, PerkinElmer Inc.) was used.

## Results

### Immunohistochemistry

**Fig. 1:**
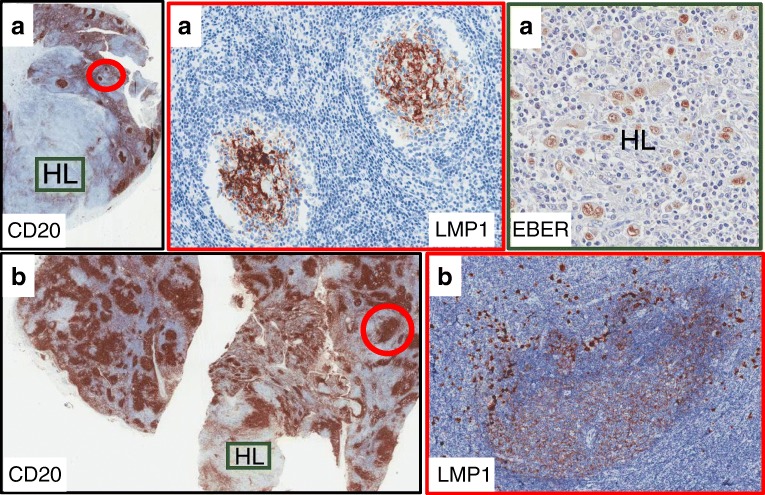
**a** Cervical lymph node of a 26-year-old male with partial involvement by EBV+ cHL NS subtype. Two small CD20+ GCs in the reactive portion of the lymph node (red circle) are intensely stained for LMP1. Stained cells have a dendritic morphology consistent with a FDC origin. In the HL lesion, numerous large cells have HRS morphology and are EBER+. **b** Cervical lymph node of an 8-year-old boy with CD20+ follicular hyperplasia and with a small focus (HL) of EBV+ cHL. In the area with follicular hyperplasia, a large LMP1+ reactive GC (red circle) is surrounded by numerous LMP1+ cells infected by EBV (higher magnification)

**Fig. 2:**
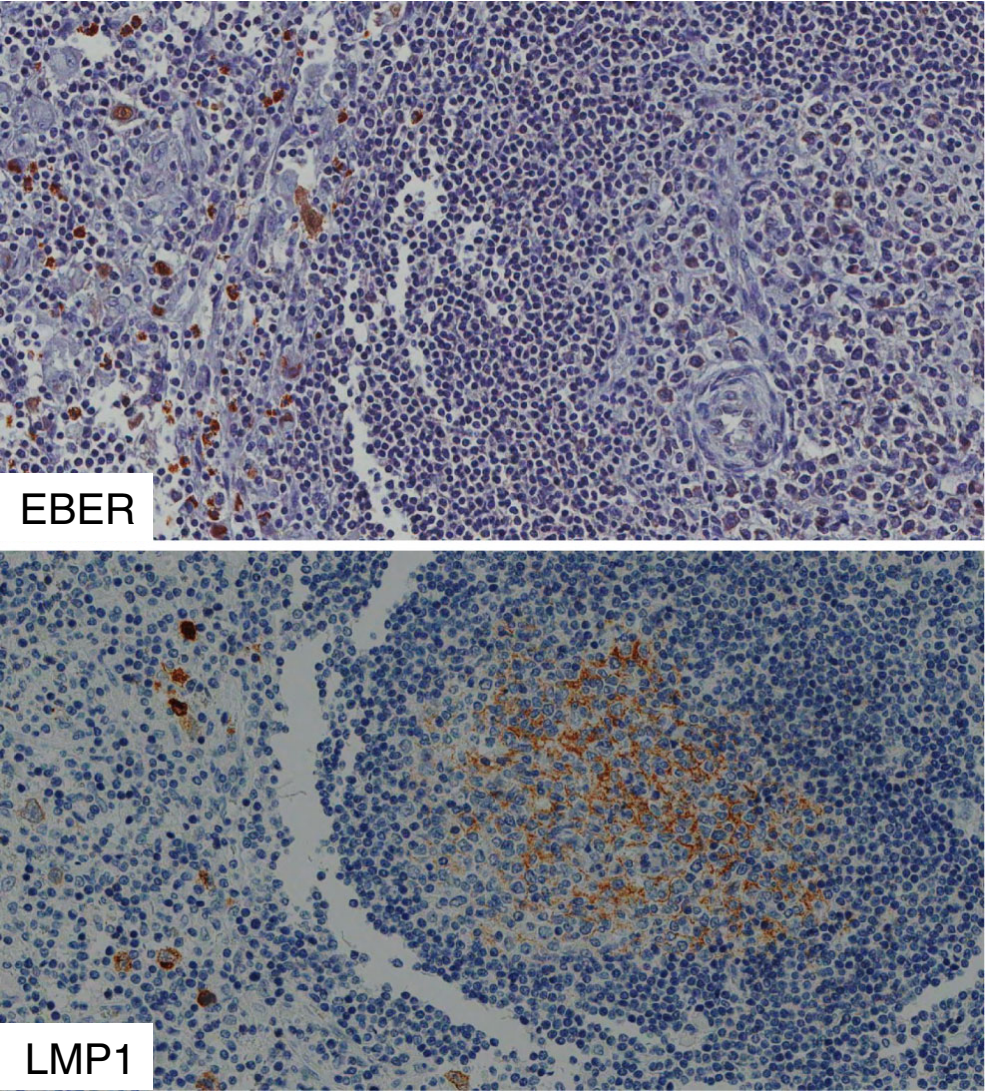
EBV+ cHL with follicular hyperplasia. EBER+/LMP1+ large cells surround a B cell follicle with FDCs positive for LMP1 and negative for EBER

Immunostaining for LMP1 protein was investigated in paraffin sections of 153 lymph nodes involved by EBV+ cHL. In all cases, the diagnosis of cHL was supported by focal or extensive lymph node effacement associated with presence of CD30+/CD15+/LMP1+ HRS cells. An unexpected finding was the observation of LMP1 staining of FDCs in reactive GC cells of 6.7% (8/120) pediatric cases and of 6.0% (2/33) adult patients (Fig. 1). LMP1+ GCs were negative for EBER, thus suggesting that FDCs were not infected by EBV; however, numerous EBER+/LMP1+ large cells surrounded and partially infiltrated the mantle zone of LMP1+ follicles (Fig. 2). Co-existence of reactive GCs and EBV+ cHL was further investigated (Table 1). It was found that variable numbers of reactive GCs were present in 22% of cases (34 of 153) and that LMP1 staining of FDCs was present in about a third of cases with reactive GCs (10 of 34). Most cases with LMP1+ GC were mixed-cellularity (MC) subtype, but some nodular sclerosis (NS) was also present. The extent of LMP1+ follicular hyperplasia was variable and was predominant in some cases, with only a limited area of the lymph node showing evidence of cHL (Fig. 1). LMP1+ GCs were not observed in other EBV-related lymphoproliferative diseases, including 125 cases of EBV+ Burkitt lymphoma [14] and 7 cases of EBV+ DLBCL [15], which were previously investigated by our group.

A survey of the clinical-pathologic features of the eight pediatric cases of EBV+ cHL with LMP1+ GCs revealed that a possible distinguishing feature was a long time interval between onset of the disease and lymph node biopsy. In fact, this time was 11 months ± 6 for the 8 cases with LMP1+ GC and 7 months ± 7 for the remaining 112 cases (*p* = 0.028; Mann Whitney test).

**Fig. 3:**
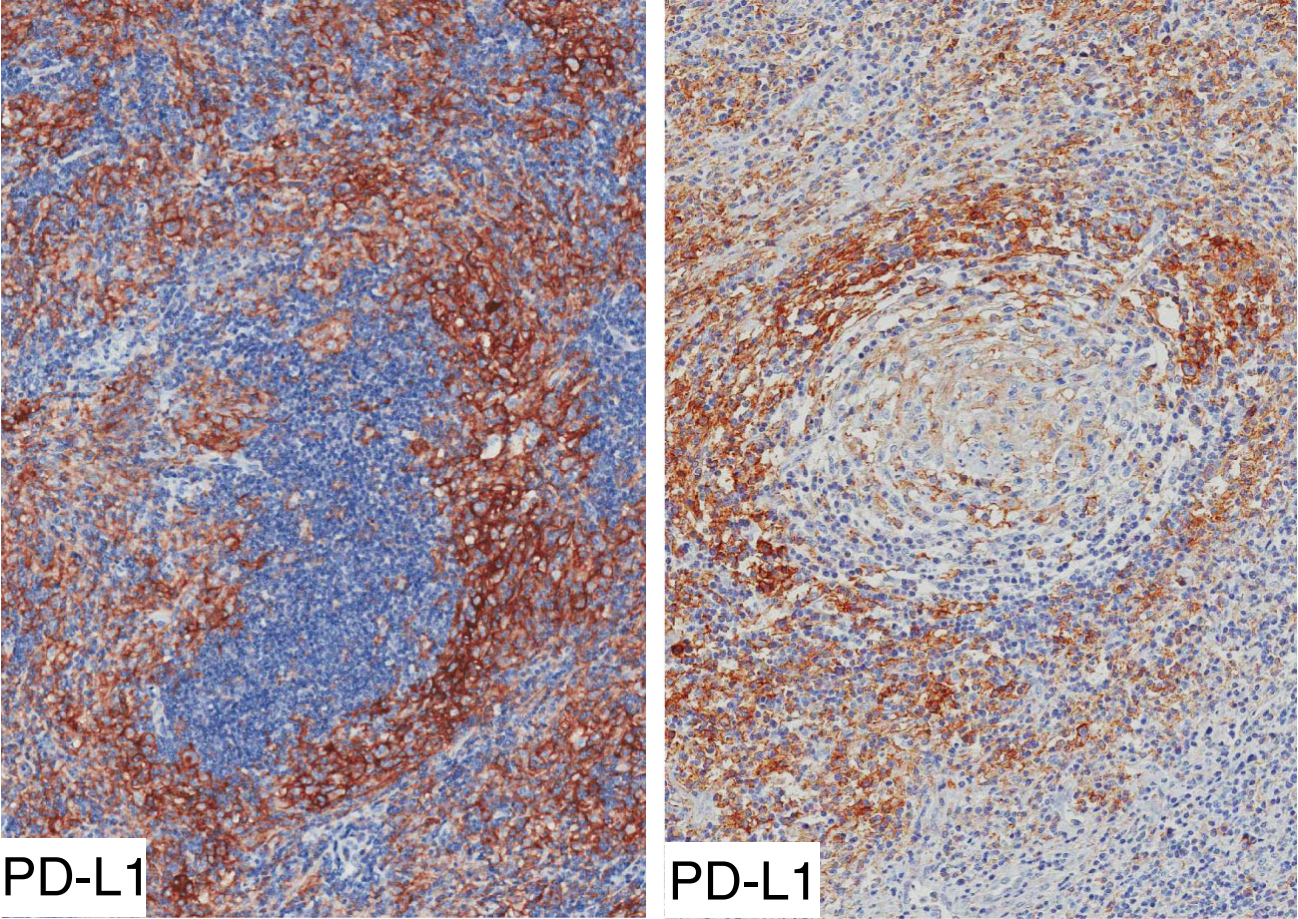
Immunostaining for PD-L1 (clone 22C3) in two cases of EBV+ cHL with reactive GCs. Numerous intensely stained cells, probably macrophages, encircle B cell follicles

LMP1 protein has an immunosuppressive role [10]. Lymph node sections were immunostained for PD-L1 and for PD1. Immunostaining for PD-L1 was detected in HRS cells and in numerous macrophages as previously reported [16, 17]. PD1+ cells were very few in HL lesions. PD-L1+ cells were mostly located in lymph node sinuses, in inter-follicular areas and occasionally in peri-follicular areas encircling B cell follicles (Fig. 3). The peri-follicular distribution of PD-L1+ cells was observed around LMP1+ and LMP1-negative GCs as well. Moreover, peri-follicular PD-L1+ cells were not observed in 19 cases of EBV+ cHL lacking LMP1+ GCs and in 13 cases of EBV-negative cHL.

### Immunofluorescence

**Fig. 4:**
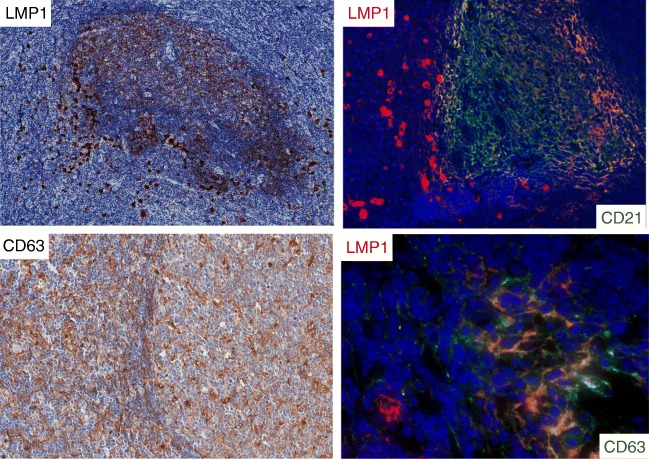
Case 1. Numerous LMP1+ large cells surround and partially infiltrate B cell follicles with LMP1+ FDCs. Double staining with immunofluorescence confirmed the presence of peri-follicular EBV-infected cells (LMP1+, red) and of CD21+ reticular FDCs (green). CD63, a marker for exosomes, was present in most stromal cells with reticular morphology inside and outside GCs. A high magnification of GC revealed the presence of double-positive cells (LMP1/CD63) with dendritic morphology

**Fig. 5:**
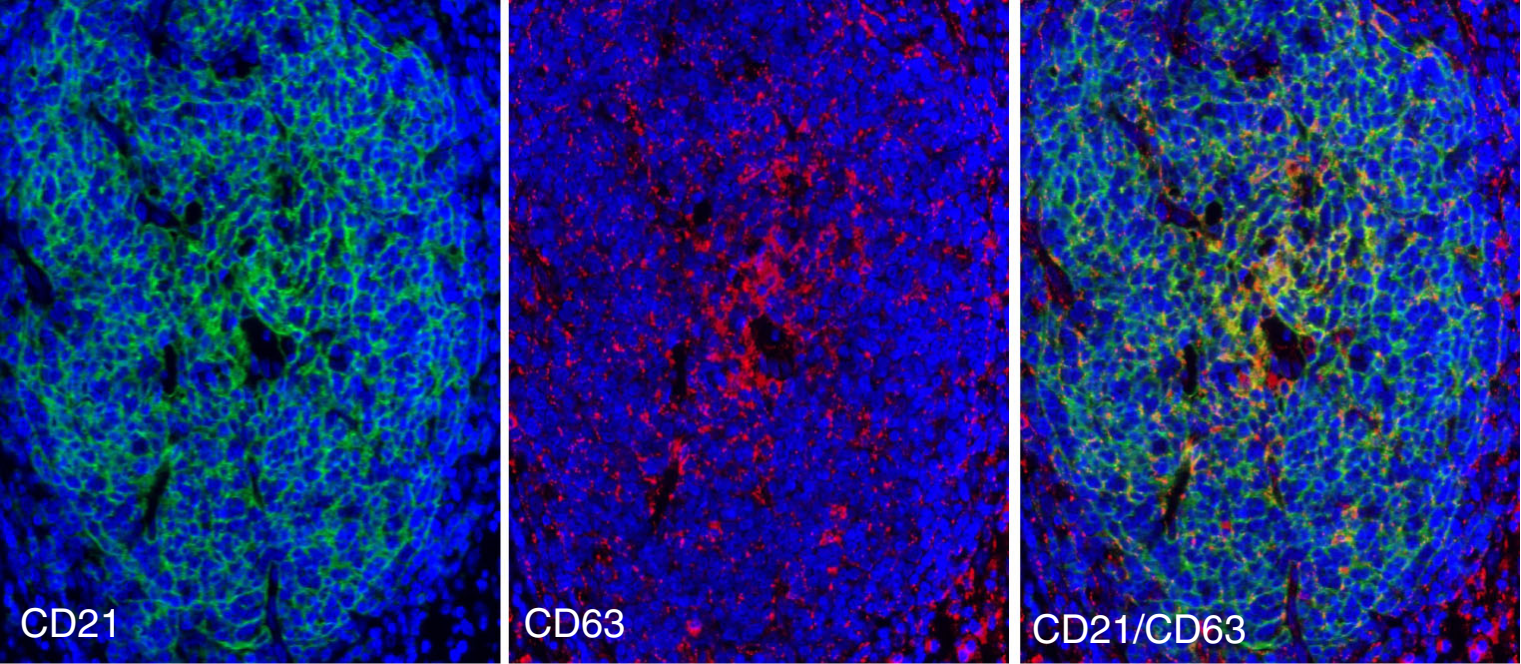
A GC positive for LMP1 was double stained for CD21 (green) and for CD63 (red). Extensive areas of co-localization of the two signals (yellow) are present, thus suggesting that CD63 protein was associated with FDCs

The relation of LMP1 protein to FDCs was further investigated using double-marker immunofluorescence. It was confirmed that numerous CD21+ FDCs were double stained for LMP1 (Fig. 4). EBV-infected cells may release LMP1 protein using exosomes [6, 7], and CD63 is an exosome-associated protein [9, 18]. Thus, lymph node sections were double stained for LMP1/CD63 or for CD21/CD63 using immunofluorescence (Figs. 4 and 5). It was found that most stromal reticular cells, inside and outside GCs, were CD63+. Furthermore, it was found that some reticular cells inside GC were double stained for LMP1/CD63 and for CD21/CD63. These observations are consistent with the possibility that LMP1 and CD63 are released by peri-follicular EBER+/LMP1+ EBV-infected cells, perhaps through exosomes, and are then captured by FDCs.

## Discussion

In the present report, we describe for the first time LMP1 protein in FDCs of reactive GCs present in cases of EBV+ cHL. LMP1+ FDCs were not infected by EBV (EBER-negative) and were present in a minority of cases (6%); moreover, their presence was independent of histological subtype, age of patients, and geographic region. A longer time interval between onset of the disease and lymph node biopsy was a distinguishing feature of some cases with LMP1+ FDCs. It can be speculated that long persistence of EBV infection in the lymph node might represent one of the events which favor accumulation of LMP1 protein in FDCs. FDCs are also known as antigen-trapping cells because of their capacity of retaining and exposing antigen on their membranes for long periods of time [19]. Thus, long-lasting accumulation of the protein prior of biopsy might be the event which favored its detection by immunohistochemistry.

It is generally assumed that EBV infection of HRS cells precedes the development of EBV+ cHL [4]. However, little is known about the EBV-induced histological modifications which precede the development of cHL. We have found that reactive GCs are present in > 20% cases of EBV+ cHL. Indeed, co-existence of follicular hyperplasia and EBV+ cHL was previously recognized [20], was defined inter-follicular cHL, and was interpreted as initial and partial involvement by cHL of a reactive lymph node. Our findings offer an alternative explanation. In fact, it can be speculated that cHL might develop in the background of a pre-existing EBV-related follicular lymphadenitis. Two lines of evidence support our interpretation. First, in some cases, LMP1+ reactive GCs were present throughout the lymph node whereas cHL was confined to a limited area (Fig. 1). Second, LMP1+ reactive GCs were often surrounded by CD30+ cells which were EBV-infected (EBER+/LMP1+). Some of them had HRS morphology, but others had a blast appearance and were of uncertain origin. Our findings are consistent with the possibility that two distinct events were occurring in the same lymph node: an EBV-related lymphadenitis, mainly involving B cell follicles, and a focal development of cHL.

It seems reasonable to postulate that LMP1 protein entrapped by FDCs was released by peri-follicular EBV-infected cells. It was previously demonstrated that EBV-infected cells may release exosomes containing LMP1 protein [6]. Exosomes are CD63+ endosomal-derived vesicles [8, 9] which transfer proteins, mRNAs, and microRNAs to neighboring or distant cells to modulate immune function, angiogenesis, cell proliferation, and tumor cell invasion. We have shown that LMP1+ FDCs were double stained for CD63 and CD21. Our observations support the view that LMP1 protein was associated with CD63+ exosomes released by peri-follicular EBV-infected cells and captured by CD21+ FDCs.

Human tumor viruses utilize exosomes for intercellular communication [7]. It can be speculated that LMP1 released by EBV-infected cells may contribute to generation of the immunosuppressive microenvironment of cHL lesions. In fact, LMP1 may induce/enhance the production of pro-inflammatory and immunomodulatory cytokines such as IL-6, IL-8, and IL-10 [5]. The relevance of the immunosuppressive microenvironment in cHL was recently highlighted by the beneficial effect of anti-PD1 immunotherapy. In the present study, we have observed that PD-L1 protein was present in HRS cells and in macrophages as previously reported [16, 17], but we have also observed that PD-L1 was highly expressed in the inter-follicular and peri-follicular cells. This topographical link may indicate that B cell follicles represent one of the sites of spreading of regulatory signals which determine the immunosuppressive background of HL lesions.
